# Study on Group Differences in Migrant Workers’ Urban Integration in China

**DOI:** 10.3389/fnint.2022.813867

**Published:** 2022-05-03

**Authors:** Haibo Li, Haitao Li, Shengyu Guo, Xuelong Fan, Feiyue Liu

**Affiliations:** ^1^Department of Economics and Management, Changsha University, Changsha, China; ^2^Bank of Leiyang Sub-Branch, Bank of China, Leiyang, China; ^3^Medical College, Hunan Normal University, Changsha, China

**Keywords:** migrant workers, urban integration, group differences, behavioral integration, psychological integration, variance analysis, optimal scaling regression analysis

## Abstract

**Objective:**

This manuscript evaluates and tests the group differences in migrant workers’ urban integration from the perspectives of individual characteristics and migration characteristics, so as to provide theoretical support and practical guidance for the government to issue more effective assistance policies.

**Methods:**

Multilevel comprehensive evaluation method and Entropy method are used to calculate the urban integration level of migrant workers, and one-way ANOVA and optimal scaling regression are used to test the group differences in migrant workers’ urban integration.

**Results:**

Based on the questionnaire data of 854 migrant workers in China, the scale of migrant workers’ urban integration has good reliability and validity. The overall level of migrant workers’ urban integration is 49.61% and there exist group differences in migrant workers’ urban integration. The impact of education level, income level, and migration time on migrant workers’ urban integration is significantly positive, whereas the impact of migration distance on migrant workers’ urban integration is significantly negative. The urban integration level of migrant workers who have family members accompanying them is higher than that of migrant workers who have no family members accompanying them. Gender, age, and marriage have no significant impact on migrant workers’ urban integration.

**Strengths and Limitations of This Study:**

This study aims to measure and test the group differences in migrant workers’ urban integration using ANOVA and optimal scaling regression. However, the shortcomings of this study are the selection of the “migrant workers’ urban integration” scale and the representativeness of the sample used in this study.

**Conclusion:**

There are group differences in migrant workers’ urban integration with different education levels, income levels, migration distances, migration times, and statuses of family members accompanying. In the policy of promoting migrant workers’ urban integration, we should accurately count the characteristics of migrant workers and give more attention to migrant workers with low education levels, low-income levels, long migration distances, short migration times, and no family accompany.

## Introduction

Migrant workers are a new type of labor force that has grown rapidly along with industrialization and urbanization since China’s reform and opening up and have made great contributions to China’s economic and social development. Statistics show that in 2020, the total number of migrant workers in China is 285.6 million, including 169.59 million migrant workers from outside the country. Social integration reflects the breadth and depth of the floating population’s participation in urban life. Only when the floating population is truly integrated into the mainstream society of the destination psychologically and behaviorally, its social integration goal can be truly achieved (Yang, 2009; Liang, 2020). As the main force of the floating population, the social integration of migrant workers is the core of promoting people-oriented new urbanization.

However, the social integration of migrant workers in China is not optimistic. With the acceleration of China’s urbanization process, Chinese farmers have not moved into cities and towns from rural areas to become real urban residents like the early western urbanization. Migrant workers have been on the edge of the city for a long time, and it has become a consensus in the academic world that it is difficult to integrate into the city. In 2020, the urbanization rate of China’s permanent resident population reached 63.89%, but the urbanization rate of the registered population was only 45%, a gap of nearly 20% points. This gap means that China’s current population of more than 280 million migrant workers is only “Half of urbanization,” which has produced tens of millions of left-behind children, elderly people, and women at the expense of three generations of migrant families. This phenomenon not only restricts the welfare of migrant workers as a large group but also affects the quality of urbanization and social harmony and stability.

Therefore, how to effectively promote the integration of migrant workers into the city, promote the orderly citizenization of the migrant agricultural population, and realize the development of “people-oriented” urbanization are important topics worthy of attention in the current academic and industrial circles. To improve the social integration levels of migrant workers in urban areas, some scholars have put forward many policy suggestions on increasing the supply of welfare public services and improving the income level of migrant workers, but these studies regard migrant workers as a whole and ignore their diversity. In practice, most of the management and services for migrant workers in China do not take into account group differences, and many policies issued by the central and local governments to improve the integration of migrant workers into cities are not targeted enough to implement fine management. In fact, migrant workers are a heterogeneous group. There are differences between migrant workers in gender, age, educational background, and other basic conditions, as well as migration characteristics such as migration distance and migration time. To clarify the urban integration of different migrant workers is the premise to accurately and effectively improve the level of migrant workers’ urban social integration. Based on this, this manuscript attempts to conduct quantitative analysis through first-hand survey data to explore the impact of group differences of Chinese migrant workers on their urban social integration and then put forward countermeasures and suggestions to improve their social integration level.

Domestic and foreign scholars’ research on urban integration is mainly based on the theory of social integration. Urban psychological integration features greatly with social and cultural differences. Foreign scholars mainly construct indicators from the perspective of social “integration,” focusing on the mutual integration of different ethnic cultures (Dustmann, 1996; Sam and Berry, 2010; Ward, 2013) reflecting the “two-way” infiltration between cultures. The most representative is Milton Gordon’s seven-dimensional measurement system (Gordon and Gordon, 1966). Chinese scholars focus on social integration mainly on the integration of domestic immigrants, especially migrant workers (Ren and Qiao, 2010; Shi and Zhu, 2014). With the continuous advancement of urbanization, urban problems are increasing, and the academic research on the urban integration of migrant workers is also increasing. To better reflect the level of migrant workers’ urban integration, some scholars have studied its evaluation system. At present, there are two main measurement orientations for the urban integration level of migrant workers as follows: one is the single overall measurement method. For example, some scholars evaluate the urban integration of migrant workers from a single factor such as the willingness of migrant workers to settle in the city and their identity (Wei et al., 2016). The second is the comprehensive scoring method, which holds that the urban integration of migrant workers is multidimensional and multilevel, and these factors should be comprehensively considered in the measurement of the urban integration level. For example, some scholars build the urban integration index system of migrant workers from economic integration, social integration, cultural integration, and psychological integration (Qin and Chen, 2014), and some scholars from market factors. Individual factors and institutional factors build the urban integration system of migrant workers (Xu and Liu, 2006), and some scholars incorporate some indicators such as the objective acceptance of the city and the establishment of a family into the urban integration comprehensive system of migrant workers (Huang and Huang, 2015).

In terms of studies on the social integration of different groups, foreign studies mainly focus on a specific group, such as the social integration of the elderly and women. For example, some studies show that gender differences exist in the social integration of international immigrants, and women give more importance to learning about new cultures and maintaining the original culture (Dion and Dion, 2001; Astondoa et al., 2020). Many other scholars have conducted studies on the social integration of the older adults (Huxhold and Fiori, 2019; Sun and Schafer, 2019; Nakagawa and Hulur, 2020), and some scholars have shown that age affects social integration through language skills (Yuksel et al., 2019). Chinese scholars have studied the group differences in migrant workers’ urban integration from the perspectives of gender, age, and education level, but the research conclusions are not consistent. For example, studies have shown that education improves social integration by improving human capital (Xiao and Zhu, 2020). However, other studies have shown that years of education and income have no significant impact on the social integration of the floating population (Wang and Zhao, 2020).

To sum up, there are still relatively few studies on the group differences in migrant workers’ social integration by scholars at home and abroad, and most of the relevant studies discuss the group differences in migrant workers’ social integration from the perspective of general individual characteristics such as gender, age, and education level, while less attention is paid to the group differences in migrant workers’ social integration from the perspective of migration characteristics. Based on the questionnaire data of 854 migrant workers, this manuscript makes a quantitative analysis of the group differences in migrant workers’ urban integration from the perspectives of individual characteristics and migration characteristics by using the methods of variance analysis and optimal scaling regression and then puts forward countermeasures and suggestions to improve their social integration level.

## Materials and Methods

### Ethical Approval

The data used in this study are from a random anonymous questionnaire. During the survey, the respondents have known and agreed to use the data for scientific research. The informed consent of participants is not required for each process carried out in this study. Therefore, this study was not applicable for review approval.

### Respondents and Investigation Methods

The subjects of this study are migrant workers who have been working in cities for more than 6 months. In terms of data acquisition, according to the principle of random sampling, Zhejiang, Guizhou, and Hunan provinces in eastern, western, and central China were selected, respectively, and several cities in each province were selected as survey sites. By taking advantage of the tradition of migrant workers going home for the Spring Festival, our research group conducted a household survey from December 2017 to March 2018. On the premise of informing migrant workers of the purpose of the survey and obtaining their consent, migrant workers were instructed to fill out the questionnaire on a one-to-one basis. Finally, 854 valid samples were collected in the following six cities: Hangzhou and Ningbo in Zhejiang Province, Guiyang and Liupanshui in Guizhou Province, and Changsha and Hengyang in Hunan Province.

### Selection of Urban Integration Evaluation Scale and Group Characteristic Variables of Migrant Workers

According to the connotation of social integration, characteristics of urban integration of migrant workers in China, experts’ advice, and the research team’s discussion, the literature analysis method is adopted to select the scale of urban integration of migrant workers and group characteristic variables. Two electronic databases for the English language and three electronic databases for the Chinese language were searched for related studies. The query time period was from 2010 to 2020.

### Test Methods of Migrant Workers’ Urban Integration Scale

The Cronbach’s α reliability coefficient was used to test the reliability of the scale. The standardized loads (STD), the component reliability (CR), and the mean variation extraction (AVE) were used to test the convergence validity of the scale. The AVE method was further used to test the discriminant validity of the scale. Exploratory factor analysis (EFA) was used to test the structural validity of the scale.

### Measurement Methods of Migrant Workers’ Urban Integration

#### Multilevel Comprehensive Evaluation Method

By considering the multilevel characteristics of the evaluation level of migrant workers’ urban integration, the multilevel comprehensive evaluation method is used to measure the level of migrant workers’ urban psychological integration. The specific methods are as follows: first, classify and calculate the urban integration of each dimension, and the level of k-class cities can be expressed as follows: *integration*_*ik*_ = ∑w_*j*_⋅*u*(*a*_*ij*_). Then, calculate the comprehensive score of each migrant worker, and the total score of urban integration of the *i*-th migrant worker can be expressed as follows: *integration*_*i*_ = ∑*p*_*k*_*integration*_*ik*_, where *p*_*k*_ = ∑*a*_*ij*_ *integration*_*i*_ is the urban integration level, *w*_*ij*_ is the weight of the *j*-th index of the *i*-th sample, and *u*(x_*ij*_) is the standardized value.

#### Calculation of Standardized Value and Weight

The entropy method is used to calculate the weight (*w*_*ij*_) of each index. The specific steps are as follows:

(1) Standardized treatment. To ensure that the standardized data are in the range of 0–1, the *max-min* method is used to standardize the index. At the same time, to make the standardized data not 0, a minimum translation is performed on the standardized data. The expression of the standardized value is as follows:

$$u\left(x_{ij}\right)=\frac{{\text{x}}_{ij}-\min\left(x_{ij}\right)}{\max\left(x_{ij}\right)-\min\left(x_{ij}\right)}+0.0001$$

(2) Calculate the index proportion. The formula for calculating the proportion of the *i*-th sample under the *j*-th index in this index is as follows: $a_{ij}=u\left(x_{ij}\right)/\sum_{i=1}^{k}u\left(x_{ij}\right)\;$.
(3) Calculate the information entropy of the index. The formula for calculating the information entropy of index *J* is as follows: $e_{j}=\sum_{i=1}^{k}a_{ij}\cdot Ina_{ij}/Ink$, where *e*_*j*_ is the information entropy of index *J*, 0 < *e*_*j*_ < 1, and *K* is the number of samples.
(4) Calculate the redundancy of information. The redundancy calculation formula of index *J* is as follows: *d*_*j*_ = 1−*e*_*j*_, where*d*_*j*_ is the redundancy of index *J*. The larger the *d*_*j*_, the more important the *j* index is.
(5) Calculate the index weight. The formula for calculating the weight of the *j*-th index is as follows: $w_{j}=d_{j}/\sum_{j=1}^{m}d_{j}$, where*w*_*j*_ is the weight of the *j*-th index, representing the contribution of the *j*-th index to the system.

### Test Methods of Group Differences in Migrant Workers’ Urban Integration

From the perspectives of individual conditions and migration characteristics, one-way ANOVA and optimal scaling regression are used to test the group differences in migrant workers’ urban integration.

#### One-Way ANOVA

One-way ANOVA is used to test the differences in migrant workers’ urban integration in different individual conditions and different migration characteristics.

#### Optimal Scaling Regression

Furthermore, the optimal scaling regression analysis was used to test the influence of individual conditions and migration characteristics on migrant workers’ urban integration.

Optimal scaling regression is an analysis method specifically used to solve the existence of multiple classification variables in the model. Under the premise that the relationship between variables after transformation becomes linear, the method quantifies the different values of classification variables. Thus, classification variables are converted into numerical types for statistical analysis. Based on the classification characteristics of explanatory variables, this manuscript adopts optimal scaling regression to empirically analyze the group differences in migrant workers’ urban integration. The optimal scaling regression analysis model is shown in the formula below:


$$Integration_{i}=\sum \alpha_{i}P_{i}+\sum \beta_{i}M_{i}+\varepsilon_{i}$$

In this type, *Integration* is explained as variable migrant workers into the city, p is individual conditions, including five variables, namely, “Gender,” “Age,” “Marriage,” “Education level,” and “Income level”, M is migration characteristic variable, including three variables, namely, “Migration distance,” “Migration time,” “family accompanying”, α_*i*_ and β_*i*_ are the coefficients, and ε_*i*_ is the error term.

## Results

### Selection of Urban Integration Evaluation Scale and Group Characteristic Variables of Migrant Workers

#### Selection of Urban Integration Evaluation Scale of Migrant Workers

The “urban integration scale of migrant workers” by scholars Li and Zhang (2020) is adopted. The scale includes two dimensions, namely, behavioral integration and psychological integration. Behavioral integration refers to the recognition and compliance of migrant workers with urban codes of conduct and customs, as well as their interaction with urban residents. It belongs to the subjective and objective behavior representation and contains four items (PI01–PI04). Psychological integration refers to migrant workers’ recognition of the inflow city, their own identity, and their psychological feelings of interaction with urban residents. It belongs to the representation of subjective consciousness and contains five items (PI05–PI09), as shown in Table 1.

**TABLE 1:** Item content and statistical description of “urban integration scale.”

Dimension	Label	Item	Min	Max	Mean	Std.
Behavioral integration	PI01	I get along well with the local people in the city	1	5	3.01	0.712
	PI02	When I encounter difficulties, I will take the initiative to ask local people around me for help	1	5	2.90	0.769
	PI03	I agree with the local way of work and life	1	5	3.07	0.709
	PI04	I have a lot of contacts with local people in the city	1	5	3.02	0.775
Psychological integration	PI05	I am very satisfied with my status in the city now	1	5	2.88	0.738
	PI06	I feel like I have the same status as the local people	1	5	3.04	0.758
	PI07	My life in the city is very happy	1	5	2.99	0.732
	PI08	I got a fair deal living in the city	1	5	3.03	0.741
	PI09	I am confident in my life in the city	1	5	3.01	0.775

#### Selection of Group Characteristic Variables of Migrant Workers

This manuscript analyzes the group differences in migrant workers’ urban integration from the perspectives of individual conditions and migration characteristics. In terms of individual conditions, this manuscript considers the differences in individual conditions of migrant workers in gender, age, marriage, education level, and income level. In terms of migration characteristics, the differences in migration characteristics mainly include migration distance, migration time, and family accompanying characteristics. From the perspective of migration distance, the regional differences of migrant workers in China are diverse, with some migration distances being shorter and some migration distances being longer. This manuscript divides the migrant workers into “inter-city non-inter-provincial migrant workers” and “inter-provincial migrant workers” according to the characteristics of cross-province regionalization. From the point of view of migration time, some work in the city for a long time, and some work for a short time. This manuscript takes 1 year and 5 years as the migration time demarcations and divides migrant workers into “short-term migrant workers” (less than 1 year), “medium-term migrant workers” (1–5 years), and “long-term migrant workers” (more than 5 years) according to migration time. From the perspective of family migration, some migrate with family accompanied, and some migrate individually. This manuscript divides migrant workers into “individual migrant workers” and “family migrant workers” according to the characteristics of family migration. This manuscript considers the differences in migration characteristics of migrant workers in these three aspects. The definition and description statistics of each variable are shown in Table 2.

**TABLE 2:** Variable definitions and descriptive statistics.

Variable	Label	Assignment	Mean	Std.
Gender	IC1	M = 1; F = 2	1.35	0.477
Age	IC2	Cenozoic = 1; The old = 2	1.54	0.499
Marriage	IC3	Unmarried = 1; Married = 2	1.70	0.459
Education Level	IC4	Primary school and below = 1; Junior high school = 2; High school = 3; High school and above = 4	2.49	0.999
Income level	IC5	1000 Y or less = 1; 1,000–3,000 (inclusive) Y = 2; 3,000–5,000 Y (inclusive) = 3; 5,000–8,000 Y (inclusive) = 4; 8,000 Y above = 5	2.84	1.004
Migration distance	MC1	Intra-provincial migration = 1; Inter-provincial migration = 2	1.41	0.492
Migration time	MC2	Less than 1 year = 1; 1–5 years = 2; More than 5 years = 3	2.06	0.749
Family accompanying	MC3	Unaccompanied = 1; With family = 2	1.54	0.499

### Reliability and Validity Test of Migrant Workers’ Urban Integration Scale

#### Test of Reliability

Cronbach’s α reliability coefficient was used to test the reliability of the scale. The results showed that the total Cronbach’s α value of the scale reached 0.87, the Cronbach’s α value of each latent variable was above 0.6, and the item-total correlation value of each variable was greater than 0.4. Cronbach’s α item deleted values are all smaller than Cronbach’s α values of their respective constructs. In summary, it indicates that the items contained in the variables of this questionnaire have good reliability.

#### Test of Structure Validity

Exploratory factor analysis results showed that the overall KMO of the questionnaire was 0.883, which passed Barlett’s spherical test (χ^2^ = 3,359.58, *P* ≤ 0.001), indicating that there existed common factors among the correlation matrices of the surveyed subjects, and it was appropriate to conduct factor analysis of the scale. Two factors were extracted from the scale, and the eigenvalues after rotation were 3.071 and 2.608, respectively, the explained variances were 34.13 and 28.98%, respectively, and the cumulative variance was 63.11%. The above results indicated satisfactory factor extraction. The two extracted factors corresponded to the two in this study, namely, behavioral integration and psychological integration.

#### Test of Convergence Validity

The results of confirmatory factor analysis (CFA) show that the STD of the two dimensions is between 0.60 and 0.90 and is significant. The CR of the two dimensions was above 0.80, and the AVE was above 0.50, indicating that the scale had high convergence validity.

#### Test of Discriminant Validity

The AVE method was further used for the discriminant validity test, and the square root AVE values of each latent variable were greater than the correlation coefficients between the latent variable and other latent variables, indicating that the two dimensions had high discriminant validity. Therefore, the two constructs have good discriminative validity. It shows that the observed variables in the scale can reflect the latent variables well, and the validity and reliability of the indicators are acceptable.

### Measurement of Migrant Workers’ Urban Integration

#### Weight Calculation

The entropy method is used to calculate the weight of each dimension and index of migrant workers’ urban integration (*w*_*ij*_), as shown in Table 2. The statistical results in Table 3 show that there is a minimal difference between the two dimensions of urban integration, both of which are approximately 50%. From the weight value of each item, the contribution of each evaluation index to the level of urbanization is relatively small. In the dimension of behavior awareness, pi04 has the largest contribution value, 32.07%, followed by pi02, with a contribution rate of 28.33%, indicating that the frequency of communication with local people and the awareness of asking local people for help are the core factors for evaluating the integration of migrant workers’ urban behavior; in the dimension of psychological identity, the contribution value of pi05 is the largest, 23.14%, indicating that urban identity is the core factor to evaluate the urban psychological integration of migrant workers. The weight values of the other six items of urban integration are more than 18%, and the difference is small, all between 18 and 21%, indicating that these items are also important indicators to evaluate the urban integration of migrant workers.

**TABLE 3:** Summary of weight calculation results of various indicators of migrant workers’ urban integration.

Dimension	Item	Information entropy	Redundancy	Weight
Behavioral integration (0.4963)	PI01	0.9890	0.0110	0.2069
	PI02	0.9849	0.0151	0.2833
	PI03	0.9899	0.0101	0.1891
	PI04	0.9829	0.0171	0.3207
Psychological integration (0.5037)	PI05	0.9860	0.0140	0.2314
	PI06	0.9888	0.0112	0.1858
	PI07	0.9886	0.0114	0.1889
	PI08	0.9887	0.0113	0.1871
	PI09	0.9875	0.0125	0.1871

#### Measurement of Migrant Workers’ Urban Integration Level

The multilevel comprehensive evaluation method is used to calculate the level values of migrant workers’ urban integration and two dimensions (*integration*_*i*_), as shown in Table 4. The overall level of migrant workers’ urban integration is 49.61%, and the levels of behavioral integration and psychological integration are 49.63 and 49.58%, respectively.

**TABLE 4:** The statistical description of migrant workers’ urban integration.

	Urban integration	Behavioral integration	Psychological integration
Mean	0.4961	0.4963	0.4958
Std.	0.1299	0.1455	0.1478

### Test of Group Differences in Migrant Workers’ Urban Integration

#### Test of One-Way ANOVA

One-way ANOVA was used to test group differences in migrant workers’ urban integration and its two dimensions. Table 5 shows that in terms of conditions of individual variables, the differences in migrant workers’ urban integration and its two dimensions among migrant workers with different education levels and different income levels are significant at the level of 1% or 5%, but the differences among migrant workers with the different gender, different age, and different marital status did not pass the significance test. In terms of migration characteristic variables, the differences in migrant workers’ urban integration among migrant workers with different migration distances are significant at the level of 1%, the differences between migrant workers with different migration times are significant at the level of 5%, and the differences between migrant workers with different characteristics of family migration are significant at the level of 5%. The results of variance analysis show that there are significant group differences in the urban integration of migrant workers with different education levels, income levels, and migration characteristics, while there are no significant group differences in the urban integration of migrant workers with the different gender, age, and marital status.

**TABLE 5:** Summary of one-way analysis of variance results.

	Urban integration		Behavioral integration		Psychological integration	
	F-value	Sig.	F-value	Sig.	F-value	Sig.
IC1	0.935	0.334	0.212	0.645	1.503	0.221
IC2	0.071	0.790	0.033	0.855	0.080	0.777
IC3	0.003	0.959	0.254	0.615	0.162	0.688
IC4	9.435***	0.000	7.388***	0.000	7.272***	0.000
IC5	5.968***	0.000	3.055**	0.016	7.536***	0.000
MC1	7.969***	0.005	12.508***	0.000	2.479*	0.084
MC2	4.267**	0.014	2.479*	0.084	4.444**	0.012
MC3	4.437**	0.035	2.482*	0.078	5.683**	0.017

***Significant at 1% level; **Significant at 5% level; *Significant at 10% level.

#### Test of Optimal Scaling Regression

Optimal scaling regression was used to test the effects of individual conditions and migration characteristics on migrant workers’ urban integration. The optimal scaling regression results in Table 6 show that the *R*^2^ of the model is 0.112, and the fitting effect of the model is acceptable. The *P*-value of ANOVA was less than 0.05, indicating that the regression model was statistically significant. The tolerance index shows that the tolerance of all variables in the model is improved after conversion, except for age, and all other variables in this manuscript are above 0.7. Therefore, it can be considered that there is no multicollinearity between variables, which further demonstrates the effectiveness of the fitting equation. However, not every variable has a role. Gender (*P* = 0.302), age (*P* = 0.937), and marriage (*P* = 0.680) did not pass the significance test, indicating that these three variables have no significant impact on migrant workers’ urban integration. All the other variables in the model passed the statistical test, indicating that education level, income level, migration distance, migration time, and family members’ accompanying statuses all have a significant impact on migrant workers’ urban integration.

**TABLE 6:** Summary of optimal scaling regression analysis results.

	Standardized coefficients		F	Sig.	Importance	Tolerance	
	Beta	Bootstrap (1000) estimate of std. error				After transformation	Before transformation
IC1	0.027	0.026	1.066	0.302	0.008	0.932	0.931
IC2	0.003	0.040	0.006	0.937	–0.003	0.653	0.619
IC3	0.010	0.025	0.170	0.680	–0.004	0.697	0.670
IC4	0.222	0.037	35.393	0.000	0.467	0.720	0.646
IC5	0.158	0.034	21.632	0.000	0.261	0.891	0.889
MC1	–0.090	0.033	7.260	0.007	0.101	0.892	0.883
MC2	0.076	0.033	5.343	0.021	0.065	0.862	0.857
MC3	0.097	0.034	8.093	0.000	0.105	0.916	0.889

*Model summary: R^2^ = 0.252; adjusted R^2^ = 0.112; ANOVA: F = 7.596; Sig. = 0.000.*

The importance index is to calculate the percentage of importance of each influence variable in the model according to the standardization coefficient and correlation coefficient. The larger the value is, the more important the variable is to the prediction of the dependent variable. According to the model, education was the most important factor (46.7%), followed by income (26.1%), and migration distance and family trailing were both more than 10%. The importance of age and marriage is negative, while the importance of gender is close to 0. This result further indicates that gender, age, and marriage have no significant impact on the migrant workers’ urban integration. The importance of migration time is 6.5%, and it is significantly positive at the level of 5%. It can be considered that migration time has an important impact on migrant workers’ urban integration.

The above regression analysis results are consistent with the previous one-way ANOVA test results, indicating that the group difference test of migrant workers’ urban integration has good reliability and robustness.

## Discussion

The urban integration of migrant workers is closely related to the development of “people-oriented” new urbanization, the citizenization of migrant workers, and the healthy development of migrant workers’ psychology and social adaptability. Based on the characteristics of migrant workers in China, the urban integration model, and common urban integration problems, this manuscript evaluates and tests the group differences in migrant workers’ urban integration from the perspectives of individual conditions and migration characteristics. The results show that (1) in terms of individual condition perspective, the impact of education level and income level on migrant workers’ urban integration is significantly positive, indicating that there are group differences in migrant workers’ urban integration with different education levels and income levels. The higher the level of education and income of migrant workers, the higher the level of urban integration. Gender, age, and marriage have no significant impact on migrant workers’ urban integration, indicating that different genders and marital statuses have no significant difference in migrant workers’ urban integration. (2) In terms of migrating feature perspective, the impact of migration distance on migrant workers’ urban integration is significantly negative, indicating that migrant workers of intra-provincial migration have a higher urban integration level than migrant workers of inter-provincial migration. The impact of migration time and family members’ accompanying statuses on migrant workers’ urban integration is significantly positive, indicating that the urban integration level of migrant workers with family members who move with them is higher than that of migrant workers who move individually, and the longer the migration time, the higher the urban integration level of migrant workers.

Based on the above research conclusions, this manuscript puts forward the following policy suggestions: (1) the characteristics of migrant workers in China should be accurately counted and a good job of information warehousing to provide information support for the management and service of migrant workers’ integration into cities should be performed; (2) population management and service measures for migrant workers should be targeted. On the basis of conventional supporting policies for migrant workers’ urban integration, more attention should be paid to migrant workers with low education level, low-income level, long migration distance, short migration time, and individual migration, and targeted policies and service rules should be issued to accurately improve their urban integration level.

The research on the group differences in migrant workers’ urban integration in this manuscript can be used by the government to evaluate the urban integration status of migrant workers. It is not only an important means for the government to monitor the urban integration status of migrant workers but also a health education tool for migrant workers to pay attention to urban integration, which can improve the urban adaptability of migrant workers. It has important application value to improve the citizenization quality of migrant workers and promote the healthy and sustainable development of people-oriented new urbanization. Nevertheless, there are still some limitations to this manuscript. First, the evaluation scale of migrant workers’ urban integration used in this study comes from the existing research results. Although this study evaluates and tests the effectiveness of the scale, whether it meets the requirements of this study needs to be further tested in the later stage of this study. Second, the data used in this study are from the questionnaire survey. Although the sample covers the west, middle, and east of China, the representativeness of the sample needs to be further verified.

## Data Availability Statement

The original contributions presented in the study are included in the article/supplementary material, further inquiries can be directed to the corresponding authors.

## Author Contributions

HL, HTL, XF, and FL: conceive, design, and perform the analysis. HTL and HL: writing – original draft of the manuscript. HL, HTL, and FL: conceptualization. HL and FL: formal analysis and methodology. HL and XF: funding acquisition and software. FL and HTL: writing – review and editing. All authors contributed to the article and approved the submitted version.

## Conflict of Interest

HTL was employed by company Bank of Leiyang Sub-Branch, Bank of China. The remaining authors declare that the research was conducted in the absence of any commercial or financial relationships that could be construed as a potential conflict of interest.

## Publisher’s Note

All claims expressed in this article are solely those of the authors and do not necessarily represent those of their affiliated organizations, or those of the publisher, the editors and the reviewers. Any product that may be evaluated in this article, or claim that may be made by its manufacturer, is not guaranteed or endorsed by the publisher.
